# A Surrogate Measure for Time-Varying Biomarkers in Randomized Clinical Trials

**DOI:** 10.3390/math10040584

**Published:** 2022-02-13

**Authors:** Rui Zhuang, Fan Xia, Yixin Wang, Ying-Qing Chen

**Affiliations:** 1 Department of Biostatistics, University of Washington, Seattle, WA 98195, USA; 2 Department of Medicine, Stanford University, Palo Alto, CA 94305, USA

**Keywords:** surrogate measure, survival settings, time-varying internal markers

## Abstract

Clinical trials with rare or distant outcomes are usually designed to be large in size and long term. The resource-demand and time-consuming characteristics limit the feasibility and efficiency of the studies. There are motivations to replace rare or distal clinical endpoints by reliable surrogate markers, which could be earlier and easier to collect. However, statistical challenges still exist to evaluate and rank potential surrogate markers. In this paper, we define a generalized proportion of treatment effect for survival settings. The measure’s definition and estimation do not rely on any model assumption. It is equipped with a consistent and asymptotically normal non-parametric estimator. Under proper conditions, the measure reflects the proportion of average treatment effect mediated by the surrogate marker among the group that would survive to mark the measurement time under both intervention and control arms.

## 1. Introduction

The HPTN (HIV Prevention Trial Network) 052 study is an HIV prevention trial conducted across several continents. The primary clinical endpoint of interest is that HIV infection is estimated to have a rate of 3–5% in modern clinical trial settings. In addition to this, the time from viral exposure to infection is long. The median infection time on average is more than one year. It is desirable to replace the clinically meaningful endpoint by an earlier and more easily accessible alternative endpoint.

A surrogate marker in clinical trials is considered to be “a laboratory measurement or physical sign used as a substitute for a clinically meaningful endpoint that measures directly how a patient feels, functions, or survives and that is expected to predict the effect of the therapy” [1]. It is considered to be valid if one could correctly conclude treatment effect on the clinical endpoint by using the marker [2,3]. In this context, how to validate surrogate markers for a clinically meaningful endpoint are especially important. Zhuang and Chen [4] review the surrogate measures in clinical research on their strengths and limitations in details.

A surrogate marker is considered to be valid if one could correctly conclude the treatment effect on the clinical endpoint by using that marker. In the language of hypothesis testing, that is, departure from the null hypothesis P(T∣Z)=P(T) is captured by departure from the null hypothesis P(S∣Z)=P(S), where Z,S, and T represent the intervention, marker, and clinical endpoint, respectively. Prentice [5] operationalized the idea to test P(T∣S,Z)=P(T∣S). The conditional independence of T and Z represents an ideal situation when the marker fully mediates the treatment effect. However, many candidate markers may only capture part of the treatment effect, so that to which extent a marker captures the treatment effect is of great practical importance. Freedman et al. [6] further extended Prentice’s criterion and evaluated the strength of surrogate markers by comparing the treatment effect with and without adjusting for the marker. For a binary endpoint T and logistic models:

$$\text{logit}\left(T|Z\right)=\mu_{1}+\beta Z,$$

$$\text{logit}\left(T|Z,S\right)=\mu_{2}+\beta_{s}Z+\phi_{z}S,$$

the proportion of treatment effect explained (PTE) is defined as $\text{PTE}=1-\beta_{s}/\beta$. However, the adjusted and unadjusted models do not hold simultaneously in general model classes [7–9], and the assumption of no interaction in the adjusted model is not necessarily true. To avoid model dependence, Wang and Taylor [9] proposed the F-measure in a general setting as F=(AA-AB)/(AA-BB), where $AA=h\left(\int_{s}g_{A}(s)dP_{A}(s)\right),\;AB=h\left(\int_{s}g_{A}(s)dP_{B}(s)\right)$, and $BB=h\left(\int_{s}g_{B}(s)dP_{B}(s)\right)$. Here, $P_{A}(s)$ and $P_{B}(s)$ are the distributions of surrogate marker S in the treatment group A and the control group B, respectively. The functions $g_{A}(s)$ and $g_{B}(s)$ are functions of the conditional distribution of the primary endpoint given S in the two groups. The functions $h(\cdot ),\;g_{A}(s)$ and $g_{B}(s)$ are chosen such that AA-BB is the desired measure of treatment effect on the primary endpoint. The F-measure framework is flexible while preserving the flavor of comparing the marginal treatment effect and adjusted treatment effect.

In this paper, we bring in the time dimension and define a generalized F-measure for time-to-event outcomes and time-varying internal surrogate markers explicitly. In Section 2, we introduce the time-varying F-measure. We show the measure can be estimated using a non-parametric estimator that is consistent and asymptotically normal. In Section 3, we give examples to visualize the change of F-measure with time and conduct Monte Carlo simulation studies to evaluate the proposed non-parametric estimation and inference. In Section 4, we apply the time-varying F-measure to an HIV prevention trial for illustration. Finally, we conclude the paper with a discussion in Section 5 and a conclusion in Section 6.

## 2. The Time-Varying F-Measure

### 2.1. Definition

We introduce the time-varying F-measure in this section. The new measure does not rely on any model assumption. In addition, it reflects Prentice’s criterion and describes the degree to which a marker captures the treatment effect on the clinical endpoints.

We consider intervention groups Z=1 and Z=0. Let T represent the time-to-event outcome and $X_{t}$ represents the value of a candidate marker measured at time point t (after randomization). The time-varying F-measure is formulated to evaluate the marker when survival status at time point c(c>t) is of primary interest. We choose $h(u)=u,\;g_{z}(x)=P\left(T\geq c|X_{t}=x,T\geq t,Z=z\right),\;P_{z}(s)=P(T\geq c|T\geq t,Z=z)$. Then, the F-measure for a time-to-event outcome T is:

$$F\left(c,t\right)=\frac{AA-AB}{AA-BB},$$

where

AA=P(T≥c|T≥t,Z=1),

BB=P(T≥c|T≥t,Z=0),

$$AB=\underset{x}{\sum }P\left(T\geq c|X_{t}=x,T\geq t,Z=1\right)P\left(X_{t}=x|T\geq t,Z=0\right).$$

It is a function of time point c when the survival status is of primary interest, and time point t when the surrogate marker is measured. The definition and estimation do not necessarily rely on any model assumption and are exempt from model misspecification.

The time-varying F-measure reflects Prentice’s criterion. Namely, the scenarios of perfect markers, in which a marker mediates all the treatment effect, lead to F(c,t)=1; the scenarios of useless markers, in which a marker does not mediate any treatment effect or is independent of intervention in the group of interest, leads to F(c,t)=0. In addition, when the treatment effect mediated by the marker is consistent with the direct treatment effect, the F-measure for a partial marker is guaranteed to be bounded within (0,1). A value outside the ideal bound indicates treatment effects via different pathways are not in the same direction so that the marker is not an appropriate surrogate. (Theoretic results are deferred to Section 2.3.)

In summary, the time-varying F-measure evaluates the relative position of the survival probability adjusted by eliminating the treatment effect on a biomarker. It serves as a model-free metric for assessing the proportion of treatment effect explained by the marker.

### 2.2. Estimation and Inference

In the time-varying F-measure, survival probabilities can be estimated by the non-parametric Kaplan–Meier estimator [10]. Under the assumption of random censoring, the conditional probability $p_{x|0}≔P\left(X_{t}=x|T\geq t,Z=0\right)$ can be estimated by the empirical distribution. Naturally, we propose a plug-in estimator for the defined time-varying F-measure:

(1) $$\hat{F}=\frac{\hat{s_{1}}-\sum_{x}\hat{s_{1x}}\cdot \hat{p_{x|0}}}{\hat{s_{1}}-\hat{s_{0}}},$$

where $\hat{s_{z}}(z=0,1)$ and $\hat{s_{1x}}$ are the Kaplan-Meier estimator for P(T≥c∣T≥t,Z=z) and $P\left(T\geq c|X_{t}=x,T\geq t,Z=1\right)$, respectively. Let $u_{z1}<u_{z2}<\ldots$ be the ordered, distinct times observed on arm $z;n_{z}(\tau )$ be the number of subjects at risk set at time τ on arm z; and $d_{z}(\tau )$ be the number of events at time τ on arm z. The Kaplan–Meier estimator of survival probabilities reads:

$$\hat{s_{z}}=\underset{t<u_{zk}\leq c}{\prod }\left(1-\frac{d_{z}\left(u_{zk}\right)}{n_{z}\left(u_{zk}\right)}\right).$$

Similarly, $s_{1x}≔Pr\left(T\geq c|X_{t}=x,T\geq t,Z=1\right)$ can be estimated by the Kaplan-Meier estimator in the strata by Z=1 and $X_{t}=x$ as:

$$\hat{s_{1x}}=\underset{t<u_{1xk}\leq c}{\prod }\left(1-\frac{d_{1x}\left(u_{1xk}\right)}{n_{1x}\left(u_{1xk}\right)}\right).$$

Under the assumption of random censoring, $p_{x|0}≔P\left(X_{t}=x|T\geq t,Z=0\right)$ can be estimated by the empirical distribution as:

$$\hat{p_{x|0}}=\frac{n_{0x}\left(t\right)}{n_{0}\left(t\right)},$$

where $n_{0x}(t)=\sum_{i=1}^{n}I\left(X_{t}=x,T\geq t,Z=0\right)$.

We show the proposed estimator converges weakly to a Gaussian process under the following regularity assumptions (Proof of the theorem is deferred to Appendix A).

**Assumption 1.** *The time* c *is in a range of* (t,τ) *for some constant* t>0,0<τ<∞ *such that* $s_{1}(\tau )s_{0}(\tau )>0$ *and* $1-\left(1-H\left(\tau_{-}\right)\right)\left(1-G\left(\tau_{-}\right)\right)<1$, *where* H *is the distribution function of time-to-event* T *and* G *is the distribution function of censoring time* U.

**Assumption 2.** *Survival probabilities* $s_{1}(\cdot )\neq s_{0}(\cdot )$ *on* (0,τ).

**Assumption 3.** *Random censoring: The censoring time* U *is independent of both the failure time* T *and time-varying covariates* $X_{t}$ *on* (0,τ).

**Theorem 1.** *𝒰nder regularity assumptions 1–3, given a time* $t,\sqrt{n_{t}}(\hat{F}(c)-F(c))$ *converges weakly to a zero-mean Gaussian process with covariance function* $E\left(\zeta (c)\zeta \left(c^{\prime }\right)\right)$ *between time points* c *and* $c^{\prime }$, *where*:

$$\zeta \left(c\right)=\frac{s_{1}-\sum_{x}p_{x|0}s_{1x}}{{\left(s_{1}-s_{0}\right)}^{2}}\cdot \eta_{0}+\frac{\sum_{x}p_{x|0}s_{1x}-s_{0}}{{\left(s_{1}-s_{0}\right)}^{2}}\cdot \eta_{1}+\frac{1}{s_{0}-s_{1}}\sum_{x}p_{x|0}\cdot \eta_{1x}+\frac{1}{s_{0}-s_{1}}\sum_{x}s_{1x}\eta_{0x^{\prime }}^{p}$$

*and*

$$\eta_{0}=-s_{0}\left(c\right)I\left(Z=0|T\geq t\right)\int_{t}^{c}\frac{dN\left(u\right)-Y\left(u\right)d\Lambda_{0}\left(u\right)}{E\left(I\left(Z=0|T\geq t\right)𝒴\left(u\right)\right)},$$

$$\eta_{1}=-s_{1}\left(c\right)I\left(Z=1|T\geq t\right)\int_{t}^{c}\frac{dN\left(u\right)-Y\left(u\right)d\Lambda_{1}\left(u\right)}{E\left(I\left(Z=1|T\geq t\right)𝒴\left(u\right)\right)},$$

$$\eta_{1x}=-s_{1}\left(c|X_{t}=x,T\geq t\right)I\left(Z=1,X_{t}=x|T\geq t\right)\int_{t}^{c}\frac{dN\left(u\right)-Y\left(u\right)d\Lambda_{1x}\left(u\right)}{E\left(I\left(Z=1,X_{t}=x|T\geq t\right)𝒴\left(u\right)\right)},$$

$$\eta_{0x}^{p}=\frac{1}{p_{0}}\left(I\left(X_{t}=x,Z=0|T\geq t\right)-p_{0x}\right)-\frac{p_{0x}}{p_{0}^{2}}\left(I\left(Z=0|T\geq t\right)-p_{0}\right).$$

*In the above equations*, N(u)≔I(T≤𝒰,T≤u) *denote the observed counting process and* 𝒴(u)≔I(T≥u,𝒰≥u) *the at-risk process. The covariance function* $E\left(\zeta (c)\zeta \left(c^{\prime }\right)\right)$ *can be consistently estimated by* $1/n_{t}\sum_{i=1}^{n_{t}}{\overset{ˆ}{\zeta }}_{i}(c){\overset{ˆ}{\zeta }}_{i}\left(c^{\prime }\right)$, *where* ${\overset{ˆ}{\zeta }}_{i}(\cdot )$ *is the sample versions of* ζ(⋅).

### 2.3. Ranges of F-Measure

#### 2.3.1. Perfect Marker

When the marker mediates the entire treatment effect, we have $P\left(T\geq c|X_{t}=x\right.,T\geq t,Z=1)=P\left(T\geq c|X_{t}=x,T\geq t,Z=0\right)$. It implies $\sum_{x}P\left(T\geq c|X_{t}=x\right.,T\geq t,Z=1)P\left(X_{t}=x|T\geq t,Z=0\right)=\sum_{x}P\left(T\geq c|X_{t}=x,T\geq t,Z=0\right)P\left(X_{t}=x|\right.T\geq t,Z=0)$, and furthermore, F(c,t)=1.

#### 2.3.2. Useless Marker

When the marker does not mediate any treatment effect, we have $P\left(T\geq c|X_{t}=x_{1}\right.,T\geq t,Z=1)=P\left(T\geq c|X_{t}=x_{2},T\geq t,Z=1\right)$; when the intervention is independent of $X_{t}$ in the risk set at time point t, we have $P\left(X_{t}=x|T\geq t,Z=1\right)=P\left(X_{t}=x|T\geq t\right.,Z=0)$. Either of the above useless marker conditions leads to $\sum_{x}P\left(T\geq c|X_{t}=x\right.,T\geq t,Z=1)P\left(X_{t}=x|T\geq t,Z=1\right)=\sum_{x}P\left(T\geq c|X_{t}=x,T\geq t,Z=1\right)P\left(X_{t}=x|\right.\;T\geq t,Z=0)$, and furthermore, F(c,t)=0.

#### 2.3.3. Partial Marker

Without loss of generality, we consider the case AA-BB>0. Theorem 2 stated below and its proof at Appendix B are naturally extendable for the counterpart case AA-BB<0. To give interpretability and links to common instances in clinical trials, we impose three mild assumptions:

**Assumption 4.** $X_{t}$ *in the treatment group and that in the control group are stochastically ordered*, $P\left(X_{t}\leq x|T\geq t,Z=1\right)≺P\left(X_{t}\leq x|T\geq t,Z=0\right)\forall x$, *or* $P\left(X_{t}\leq x|T\geq t\right.,Z=1)≻P\left(X_{t}\leq x|T\geq t,Z=0\right)\forall x$.

**Assumption 5.** $P\left(T\geq c|X_{t}=x,T\geq t,Z=z\right)$ *is monotone with* x *in the same direction for any given* z.

**Assumption 6.** $P\left(T\geq c|X_{t}=x,T\geq t,Z=z\right)$ *is monotone with* z *in the same direction for any given* x.

In addition, we formulate three conditions:

C1. $P\left(T\geq c|X_{t}=x,T\geq t,Z=1\right)-P\left(T\geq c|X_{t}=x,T\geq t,Z=0\right)>0$.
C2. $P\left(X_{t}\leq x|T\geq t,Z=1\right)≺P\left(X_{t}\leq x|T\geq t,Z=0\right)\forall x$ and $P\left(T\geq c|X_{t}=x\right.,T\geq t,Z=1)$ is increasing with x.
C3. $P\left(X_{t}\leq x|T\geq t,Z=1\right)≻P\left(X_{t}\leq x|T\geq t,Z=0\right)\forall x$ and $P\left(T\geq c|X_{t}=x\right.,T\geq t,Z=1)$ is decreasing with x.

**Theorem 2.** *With Assumptions 4–6, if Condition C1 is satisfied, then* F<1; *if either Condition C2 or C3 is satisfied, then* F>0.

### 2.4. Causal Interpretation

The F-measure is closely related with the concept of natural indirect effect, which is defined in the counterfactual framework [11,12]. We formulate Theorem 3 revealing the link with detailed proof in Appendix C.

**Assumption 7.** $\left\{T\left(Z=1,X_{t}=x\right),X_{t}(Z=1)\right\}⊥\left\{T\left(Z=0,X_{t}=x\right),X_{t}(Z=0)\right\}$.

**Assumption 8.** $Z⊥\left\{T\left(Z=z,X_{t}=x\right),X_{t}(Z=z)\right\}$.

**Assumption 9.** $X_{t}(Z=1)⊥T\left(Z=1,X_{t}=x\right)|T\geq t,Z=1$.

**Theorem 3.** *Under Assumptions 7–9, it holds that:*

$$F=\frac{P\left(T\left(1\right)\geq c|T\left(1\right)\geq t,T\left(0\right)\geq t\right)-P\left(T\left(1,X_{t}=X_{t}\left(0\right)\right)\geq c|T\left(1\right)\geq t,T\left(0\right)\geq t\right)}{P\left(T\left(1\right)\geq c|T\left(1\right)\geq t,T\left(0\right)\geq t\right)-P\left(T\left(0\right)\geq c|T\left(1\right)\geq t,T\left(0\right)\geq t\right)}.$$

For the subgroup of T(1)≥t,T(0)≥t, F-measure’s numerator describes the natural indirect effect mediated by the surrogate marker while the denominator describes the average treatment effect. The ratio reflects the proportion of the average treatment effect mediated by the surrogate measure (in the sense of natural direct effect) for the subgroup surviving to marker measurement anyway. However, we also note that the causal interpretation does not apply in general [13].

## 3. Numerical Studies

To assess the proposed surrogate measure, we conduct numerical studies motivated by the HIV Prevention Trial Network. The plasma HIV-1 viral load represents the degree of viral burden and is believed to play a crucial role in mediating the benefit of antiretroviral therapy (ART) on HIV-related disease progression and transmission. We consider a viral load measurement dichotomized by a threshold of 1000 copies per cubic millimeter as the biomarker of interest. In a hypothetical scenario, participants have some HIV-1 exposure at the enrollment. The viral load level may increase fast in the follow-up while an effective intervention could delay the virus proliferation and further suspend the failure time. We express the above scenario in the following mathematical models. The dichotomous viral load level at time t is modeled as $X_{t}=I\left(t\geq t_{s}\right)$, where $t_{s}$ denotes the time when one’s viral load shifts from level 0 to 1 after enrollment. We assume $t_{s}$ follows an exponential distribution with mean $\mu_{z}$ in intervention group z, and a time-varying Cox-Weibull model:

(2) $$h\left(t|X_{t},Z\right)=h_{0}\left(t\right)\text{exp}\left(b_{1}Z+b_{2}X_{t}\right),$$

(3) $$h_{0}\left(t\right)=\lambda vt^{v-1},$$

where Z is Bernoulli with success probability of 0.5.

### 3.1. Numerical Examples

**Figure 1.**
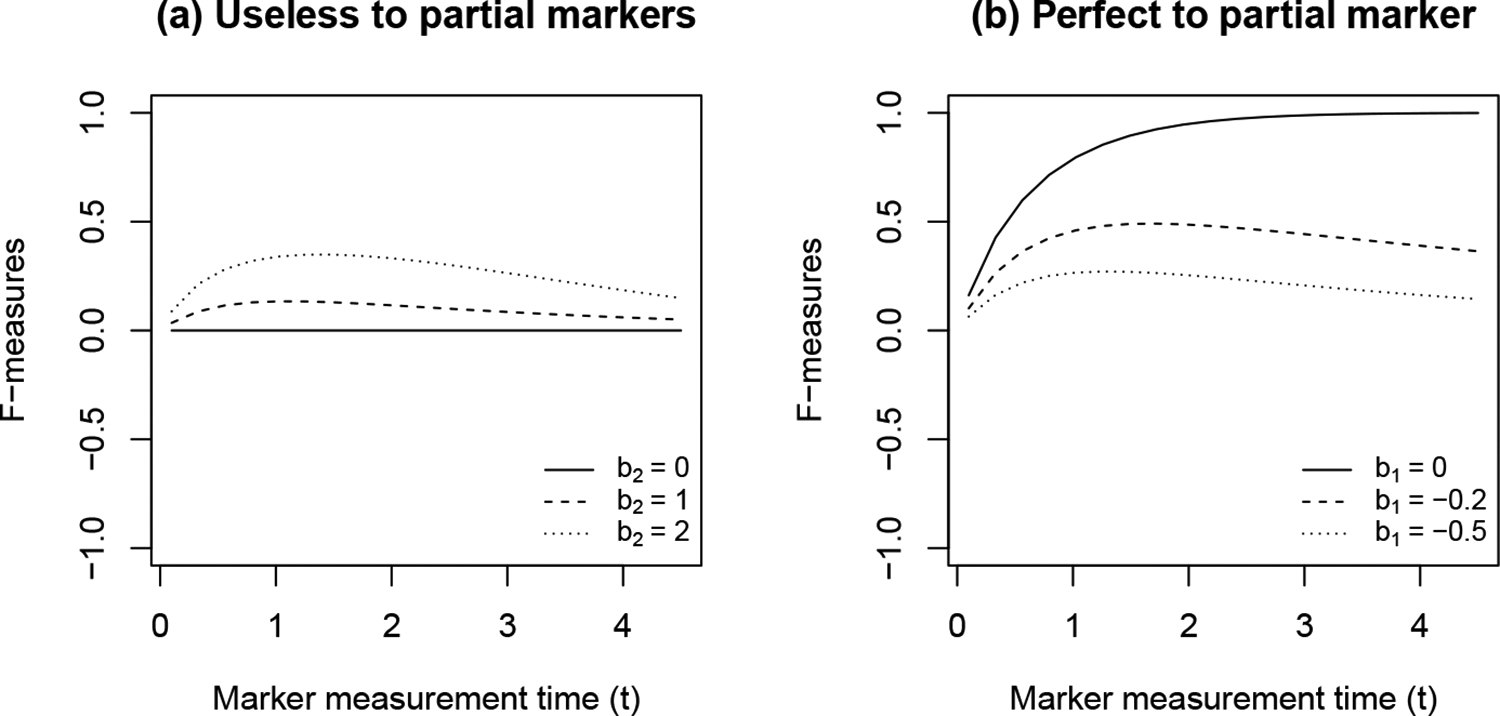
F-measure curves describing the surrogacy level for survival status at Year 5.

In Figure 1, we explore the numerical behavior of the time-varying F-measure under the motivation scenario described above. In particular, we assume Model (2) with a constant baseline hazard where $h_{0}(t)=0.2$. Without loss of generality, we assume $b_{1}\leq 0,b_{2}\geq 0$, and c=5. With the model assumption, the F-measure has a closed-form formula with details in the Appendix D. Figure 1a has $b_{1}=-1,t_{0}=0.5,t_{1}=2$, varying $b_{2}$ visualized F-measures curves from an useless marker with $b_{2}=0$ to partial markers with $b_{2}>0$. Figure 1b has $b_{2}=1,t_{0}=0.5,t_{1}=2$, varying $b_{1}$ gives the F-measure curves from a perfect marker with $b_{1}=1$ to partial markers with $b_{1}<0$.

### 3.2. Monte–Carlo Simulation

In this section, we describe our Monte–Carlo simulation to evaluate the proposed non-parametric estimator. We generate failure times for each z-group based on a closed-form approach described in Austin [14]: First, a random value u is generated from the Uniform (0, 1) distribution and the subject-specific shift time $t_{s}$ is generated from an exponential distribution with mean $\mu_{z}$; second, $\text{if}-\text{log}\left(u\right)<\lambda \;\text{exp}\left(b_{1}z\right)t_{s}^{v}$, we let failure time $T={\left(-\text{log}(u)/\left(\lambda \;\text{exp}\left(b_{1}z\right)\right)\right)}^{1/v}$; otherwise, $T=\left(\left(-\text{log}\left(u\right)-\lambda \;\text{exp}\left(b_{1}z\right)t_{s}^{v}+\right.\right.\;{\left.\left.\lambda \;\text{exp}\left(b_{2}\right)\text{exp}\left(b_{1}z\right)t_{s}^{v}\right)/\left(\lambda \;\text{exp}\left(b_{2}\right)\text{exp}\left(b_{1}z\right)\right)\right)}^{1/v}$. In addition, we generate the censoring times from Uniform (0,τ), in which τ is chosen to give a censoring rate of 20%. The censoring is independent of the failure time T, the covariates Z and $X_{t}$.

Table 1 summarizes the simulation results. We consider the scale parameter v to be 0.8, 1, and 1.2, representing when the hazard is decreasing, constant, and increasing with time, respectively. We are interested in the surrogacy level of the t-th year marker measurement for the treatment effect on the c-th year survival probability. For each setting of v, we choose c=5 (years) and t=0.25,0.5,1,2 (years). We show typical scenarios when a surrogate marker is perfect, useless, or partial. A perfect surrogate marker explains all the treatment effect on the clinical endpoint, i.e., $b_{1}=0$; an useless maker is conditionally independent of the failure time given Z, i.e., $b_{2}=0$; a partial marker, the most common scenario in practice, is beyond the above extreme situations. Without loss of generality, we consider a treatment delaying the failure time by both directly affecting the clinical endpoint and suppressing a harmful marker. That is, $b_{1}\leq 0,$ $b_{2}\geq 0$, and $\mu_{0}\leq \mu_{1}$. Specifically, here are the configurations for the three scenarios in Table 1: (1) A perfect marker: $\lambda =0.02,b_{1}=0,b_{2}=3,t_{0}=3$ months, $t_{1}=30$ months; (2) a useless marker: $\lambda =0.3,b_{1}=-1,b_{2}=0,t_{0}=3$ months, $t_{1}=30$ months; and (3) a partial marker: $\lambda =0.2,b_{1}=-0.5,b_{2}=0.5,t_{0}=3$ months, $t_{1}=30$ months. We replicate 1000 times with 20,000 subjects. Under a large sample size, the estimator is unbiased; its variance accurately reflects the sampling variation; the coverage of 95% Wald-type confidence intervals is close to the nominal probability. One limitation for the non-parametric estimator is its lack of efficiency, which is the price for avoiding model misspecification.

## 4. Data Analysis

We apply the proposed time-varying F-measure to the HIV Prevention Trial Network (HPTN) 052 study [15]. The study enrolled 1763 serodiscordant couples in which one participant was HIV-positive, and the other was HIV-negative. The HIV-positive patients were randomly assigned to receive either immediate or delayed ART in a 1:1 ratio. Patients on the delayed arm started ART when two consecutive CD4+ cell count measurements fell below 250 per cubic millimeter or an indicator of AIDS developed. The study monitored the earlier occurrence of severe clinical outcomes in HIV-positive patients or HIV transmission to HIV-negative partners as a key endpoint. It is believed that plasma viral load mediates the effect of ART on HIV-associated disease progression and transmission [16].

In this application, we consider the plasma viral load as a candidate marker and evaluate its surrogacy level on the composite monitoring endpoint in a 3-year follow-up. To explain the idea in a simple way, we dichotomize the viral load using a threshold of 1000 copies per cubic millimeter. More specifically, we set the marker value to be 1 for a viral load greater than 1000. We estimate the time-varying F-measure for the viral load measured at each of the 2nd to 7th quarter after randomization. Table 2 shows results of the application. Comparing the prevalence of a high viral load between the two arms reveals that ART was very effective in suppressing viral proliferation. In addition, a low viral load significantly decreases the hazard of the composite endpoint on the immediate arm before the treatment effect kicks in on the delayed arm. The time-varying F-measure gradually increases until reaching its maximum at the 6th quarter. This temporal pattern reflects the fact that the surrogacy level is a combination of the treatment effect on the marker and the marker effect on the clinical endpoint. On the one hand, it takes time to realize the effect of viral load suppression. On the other hand, as an increasing number of patients on the delayed arm began ART, the difference in marker distribution between two arms become smaller. The time-varying F-measure correctly reflects the temporal pattern and the biological mechanism of ART.

## 5. Discussion

In this paper, we consider a definition of time-varying F-measure based on three aspects. First, there is the question of whether there is a sound interpretation for comparisons. Second, do the typical marker types, such as perfect or useless markers, correspond to reasonable values. Third, is the defined F-measure model-free and equipped with a non-parametric estimation? Guided by the three questions, we define the time-varying F-measure in Section 2. In addition, we explore two alternative definitions. Both of them do not conduct an appropriate comparison. With $g_{z}(x)=P\left(T\geq c|X_{t}=x,Z=z\right)$, the F-measure can be defined as:

$$F\left(c,t\right)=\frac{P\left(T\geq c|Z=1\right)-\sum_{x}P\left(T\geq c|X_{t}=x,Z=1\right)P\left(X_{t}=x|Z=0\right)}{P\left(T\geq c|Z=1\right)-P\left(T\geq c|Z=0\right)}.$$

When the availability of an internal marker depends on the failure time (e.g., event is death-related), $X_{t}$ should include “not applicable” as a possible value for subjects with T<t. In this case, $P\left(X_{t}=x|Z=1\right)-P\left(X_{t}=x|Z=0\right)$ is determined by both the treatment effect on the marker and that on the primary endpoint. Compared to P(T≥c∣Z=1), the adjusted survival probability actually removes a portion of the direct treatment effect. This definition does not reflect the proportion of treatment effect explained by the surrogate marker in general. With $g_{z}(x)=h\left(c|X_{t}=x,Z=z\right)$, the F-measure can be defined as:

$$F\left(c,t\right)=\frac{h\left(c|Z=1\right)-\sum_{x}h\left(c|X_{t}=x,Z=1\right)P\left(X_{t}=x|T\geq c,Z=0\right)}{h\left(c|Z=1\right)-h\left(c|Z=0\right)}.$$

A closer look at the marker distribution $P\left(X_{t}=x|T\geq c,Z=z\right)$ reveals that:

$$P\left(X_{t}=x|T\geq c,Z=z\right)={\left(1+\underset{y\neq x}{\sum }\frac{P\left(T\geq c|X_{t}=y,T\geq t,Z=z\right)P\left(X_{t}=y|T\geq t,Z=z\right)}{P\left(T\geq c|X_{t}=x,T\geq t,Z=z\right)P\left(X_{t}=x|T\geq t,Z=z\right)}\right)}^{-1}.$$

If there is no interaction between the marker and intervention, the independence of $X_{t}$ and Z in the risk set at time point t could translate to the independence at time point c. In other words, only if $P\left(T\geq c|X_{t}=y,T\geq t,Z=1\right)/P\left(T\geq c|X_{t}=y\right.,\;T\geq t,\text{Z}=1)=P\left(T\geq c|X_{t}=y,T\geq t,Z=0\right)/P\left(T\geq c|X_{t}=y,T\geq t,Z=0\right)$, then $P\left(X_{t}=x|T\geq c,Z=1\right)=P\left(X_{t}=x|T\geq c,Z=0\right)$ is equivalent to $P\left(X_{t}=y|T\geq \right.\;t,\text{Z}=z)/P\left(X_{t}=x|T\geq t,Z=z\right)$ and is constant with z. Assumption of no interaction is, unfortunately, necessary for the appropriateness of the definition with hazard functions. As a contrast, the time-varying F definition introduced in Section 2 has a sound interpretation, reasonable ranges, and model-free definition and estimation. Moreover, numerical studies and practical data analysis verify the measure’s numerical behavior.

The time-varying F-measure is a generalization of the PTE [6] and F-measure [9]. All three measures are quantitative ones based on the qualitative Prentice Criterion [5]. While Prentice Criterion tests P(T∣S,Z)=P(T∣S) and requires a surrogate marker to capture the treatment effect fully, the three quantitative measures compare the treatment effects unadjusted and adjusted by the marker distribution on the treatment arm. Beyond the similarities, PTE is defined for binary endpoints and relies on logistic regressions for definition and estimation; the F-measure is a model-free version of PTE, however it does not cover how to assess surrogate markers for time-to-event outcomes. The time-varying F-measure brings in the time dimension and extends the measure for time-to-event outcomes in survival settings.

## 6. Conclusions

This paper introduces a generalized proportion of treatment effect for survival settings, called the time-varying F-measure. Without relying on any model assumption, the measure reflects the proportion of the average treatment effect mediated by the surrogate marker. In addition, the paper introduces a non-parametric estimator to maximize the measure’s model-free characteristics. One limitation of the current estimation method is its lack of efficiency, which can be a future research direction. We applied the generalized F-measure to assess the viral load as a surrogate marker for HIV progression and transmission in the HPTN052 study. The time-varying F-measure increased from 0.18 in the 2nd quarter after randomization and reached 1.12 in the 6th quarter. It correctly captured the temporal pattern and biological mechanism of how ART regulates HIV progression and transmission by suppressing viral replication.

## Figures and Tables

**Table 1.** Simulation results under Cox-Weibull distribution. The sample size of the study is 20,000 subjects and the coverage probability is obtained by 1000 replicates.

c = 5, v = 0.8						
Scenario	Marker time^a^	True value	Bias	Sampling SE	Mean of SE	Coverage
	0.25	0.747	0.003	0.052	0.051	0.941
Perfect	0.5	0.932	0.002	0.054	0.055	0.956
	1	0.995	0.003	0.059	0.059	0.953
	2	1.000	0.007	0.081	0.080	0.948
	0.25	0.000	0.001	0.034	0.034	0.948
Useless	0.5	0.000	0.001	0.031	0.030	0.951
	1	0.000	0.001	0.023	0.023	0.949
	2	0.000	0.001	0.017	0.016	0.944
	0.25	0.197	0.003	0.051	0.051	0.955
Partial	0.5	0.229	0.001	0.047	0.047	0.953
	1	0.213	0.002	0.038	0.037	0.952
	2	0.167	0.003	0.030	0.030	0.957
c = 5, v = 1						
Scenario	Marker time^a^	True value	Bias	Sampling SE	Mean of SE	Coverage
	0.25	0.743	0.002	0.043	0.044	0.951
Perfect	0.5	0.931	0.002	0.044	0.046	0.954
	1	0.995	0.002	0.047	0.048	0.949
	2	1.000	0.004	0.062	0.063	0.945
	0.25	0.000	0.001	0.033	0.034	0.964
Useless	0.5	0.000	0.000	0.030	0.030	0.958
	1	0.000	0.000	0.022	0.022	0.954
	2	0.000	0.001	0.015	0.016	0.954
	0.25	0.204	0.002	0.051	0.051	0.951
Partial	0.5	0.241	0.000	0.046	0.046	0.953
	1	0.228	0.000	0.036	0.036	0.960
	2	0.181	0.001	0.028	0.029	0.951
c = 5, v = 1.2						
Scenario	Marker time^a^	True value	Bias	Sampling SE	Mean of SE	Coverage
	0.25	0.742	0.002	0.036	0.036	0.949
Perfect	0.5	0.930	0.002	0.036	0.037	0.956
	1	0.995	0.001	0.037	0.038	0.952
	2	1.000	0.003	0.049	0.048	0.940
	0.25	0.000	0.001	0.037	0.037	0.953
Useless	0.5	0.000	0.000	0.032	0.032	0.955
	1	0.000	0.000	0.023	0.023	0.943
	2	0.000	0.000	0.016	0.016	0.953
	0.25	0.219	0.003	0.055	0.054	0.948
Partial	0.5	0.262	0.000	0.049	0.049	0.956
	1	0.252	0.000	0.037	0.038	0.947
	2	0.203	0.001	0.030	0.030	0.952

a The time point (year) measuring the marker.

**Table 2.** Application to an HIV prevention trial HPTN 052. The proposed time-varying F-measure captures the proportion of treatment effect explained by the plasma HIV-1 viral load.

Marker Time^a^	Delayed ART Arm		Immediate ART Arm		F-Measure	
	Prevalence of Viral Load ≥ 1000	Hazard Ratio^b^	Prevalence of Viral Load ≥ 1000	Hazard Ratio^b^	Estimator	95% CI
2	0.88	1.39	0.08	2.20	0.18	−0.03, 0.39
3	0.88	0.94	0.08	3.21*	0.41	0.13, 0.70
4	0.87	1.00	0.09	4.49*	0.52	0.09, 0.95
5	0.85	1.59	0.08	5.59*	0.72	0.10, 1.34
6	0.81	2.51*	0.07	4.49*	1.12	−0.42,2.67
7	0.75	2.11	0.08	6.55*	0.81	−0.92,2.54

a The time point (quarter) when plasma HIV-1 viral load was measured.

b Hazard ratio between groups with a viral load higher and lower than 1000 copies per cubic millimeter.

Significant results at the level of 0.05 are marked with *.

## Appendix A. Proof of Theorem 1

**Proof.** The proof consists of three steps. First, we decompose $\sqrt{n_{t}}(\hat{F}-F)$ as multiple empirical processes. Second, the convergence of each empirical process is derived. Third, we combine the asymptotic results and conclude the proof.

*Step 1:* We first write $\sqrt{n_{t}}(\hat{F}-F)$ as:

$$\sqrt{n_{t}}\left(\hat{F}-F\right)=\sqrt{n_{t}}\left(\hat{F}\left(\hat{p_{x|0}}\right)-F\left(\hat{p_{x|0}}\right)\right)+\sqrt{n_{t}}\left(F\left(\hat{p_{x|0}}\right)-F\left(p_{x|0}\right)\right),$$

and tackle the parts one by one. For the first part, we plug in the estimator (1) and obtain:

$$\sqrt{n_{t}}\left(\hat{F}\left(\hat{p_{x|0}}\right)-F\left(\hat{p_{x|0}}\right)\right)=\sqrt{n_{t}}\left(\frac{\hat{s_{1}}-\sum_{x}\hat{s_{1x}}\cdot \hat{p_{x|0}}}{\hat{s_{1}}-\hat{s_{0}}}-\frac{s_{1}-\sum_{x}s_{1x}\hat{p_{x|0}}}{s_{1}-s_{0}}\right).$$

Rearranging the terms yields:

$$\sqrt{n_{t}}\left(\hat{F}\left(\hat{p_{x|0}}\right)-F\left(\hat{p_{x|0}}\right)\right)\;=\frac{\sqrt{n_{t}}}{\left(\hat{s_{1}}-\hat{s_{0}}\right)\left(s_{1}-s_{0}\right)}\left(s_{1}\left(\hat{s_{0}}-s_{0}\right)-s_{0}\left(\hat{s_{1}}-s_{1}\right)-\underset{x}{\sum }\hat{p_{x|0}}\left(s_{1}-s_{0}\right)\left(\hat{s_{1x}}-s_{1x}\right)-\underset{x}{\sum }\hat{p_{x|0}}s_{1x}\left(\hat{s_{0}}-s_{0}\right)+\underset{x}{\sum }\hat{p_{x|0}}s_{1x}\left(\hat{s_{1}}-s_{1}\right)\right).$$

Then we collect the terms and write the equation in the form of $\sqrt{n_{t}}\left(\hat{s_{0}}-s_{0}\right),\sqrt{n_{t}}\left(\hat{s_{1}}-\right.\left.s_{1}\right)$ and $\sqrt{n_{t}}\left(\hat{s_{1x}}-s_{1x}\right)$ as:

$$\sqrt{n_{t}}\left(\hat{F}\left(\hat{p_{x|0}}\right)-F\left(\hat{p_{x|0}}\right)\right)\;=\frac{s_{1}-\sum_{x}\hat{p_{x|0}}s_{1x}}{\left(\hat{s_{1}}-\hat{s_{0}}\right)\left(s_{1}-s_{0}\right)}\cdot \sqrt{n_{t}}\left(\hat{s_{0}}-s_{0}\right)+\frac{\sum_{x}\hat{p_{x|0}}s_{1x}-s_{0}}{\left(\hat{s_{1}}-\hat{s_{0}}\right)\left(s_{1}-s_{0}\right)}\cdot \sqrt{n_{t}}\left(\hat{s_{1}}-s_{1}\right)+\underset{x}{\sum }\frac{\hat{p_{x|0}}\left(s_{0}-s_{1}\right)}{\left(\hat{s_{1}}-\hat{s_{0}}\right)\left(s_{1}-s_{0}\right)}\cdot \sqrt{n_{t}}\left(\hat{s_{1x}}-s_{1x}\right).$$

Similarly, we write the second part as:

$$\sqrt{n_{t}}\left(F\left(\hat{p_{x|0}}\right)-F\left(p_{x|0}\right)\right)\;=\sqrt{n_{t}}\left(\frac{s_{1}-\sum_{x}s_{1x}\hat{p_{x|0}}}{s_{1}-s_{0}}-\frac{s_{1}-\sum_{x}s_{1x}p_{x|0}}{s_{1}-s_{0}}\right)=\frac{1}{s_{0}-s_{1}}\underset{x}{\sum }s_{1x}\cdot \sqrt{n_{t}}\left(\hat{p_{x|0}}-p_{x|0}\right).$$

*Step 2:* In this step, we derive the convergence of each empirical process. Under the assumption of random censoring, Kaplan–Meier estimator $\hat{S}(c)$ satisfies [17]:

$$\sqrt{n_{t}}\left(\hat{S}\left(c\right)-S\left(c\right)\right)=\sqrt{n_{t}}\left({𝒫}_{n}-𝒫\right)\left(-S\left(c\right)\int_{0}^{c}\frac{dM\left(u\right)}{EY\left(u\right)}\right)+o_{p}\left(1\right),$$

where $M(u)≔N(u)-\int_{0}^{u}Y(a)d\Lambda (a)$ represents the counting process martingale. Therefore, the Kaplan–Meier estimator $\hat{s_{0}},\hat{s_{1}}$, and $\hat{s_{1x}}$ satisfy:

$$\sqrt{n_{t}}\left(\hat{s_{0}}\left(c\right)-s_{0}\left(c\right)\right)\;\overset{d}{=}\sqrt{n_{t}}\left({𝒫}_{n}-𝒫\right)\left(-s_{0}\left(c\right)I\left(Z=0|T\geq t\right)\int_{t}^{c}\frac{dN\left(u\right)-Y\left(u\right)d\Lambda_{0}\left(u\right)}{E\left(I\left(Z=0|T\geq t\right)Y\left(u\right)\right)}\right),\;\sqrt{n_{t}}\left(\hat{s_{1}}\left(c\right)-s_{1}\left(c\right)\right)\;\overset{d}{=}\sqrt{n_{t}}\left({𝒫}_{n}-𝒫\right)\left(-s_{1}\left(c\right)I\left(Z=1|T\geq t\right)\int_{t}^{c}\frac{dN\left(u\right)-Y\left(u\right)d\Lambda_{1}\left(u\right)}{E\left(I\left(Z=1|T\geq t\right)Y\left(u\right)\right)}\right),\;\sqrt{n_{t}}\left(\hat{s_{1x}}\left(c\right)-s_{1x}\left(c\right)\right)\;\overset{d}{=}\sqrt{n_{t}}\left({𝒫}_{n}-𝒫\right)\;\left(-s_{1x}\left(c\right)I\left(Z=1,X_{t}=x|T\geq t\right)\int_{t}^{c}\frac{dN\left(u\right)-Y\left(u\right)d\Lambda_{1x}\left(u\right)}{E\left(I\left(Z=1,X_{t}=x|T\geq t\right)Y\left(u\right)\right)}\right),$$

where I(⋅) is an indicator function for subjects’ group.

Next we write $\sqrt{n_{t}}\left(\hat{p_{x|0}}-p_{x|0}\right)$ in the form of:

$$\sqrt{n_{t}}\left(\hat{p_{x|0}}-p_{x|0}\right)=\sqrt{n_{t}}\left(\hat{P}\left(X_{t}=x|T\geq t,\text{Z}=0\right)-P\left(X_{t}=x|T\geq t,\text{Z}=0\right)\right)\;=\sqrt{n_{t}}\left(\frac{\hat{P}\left(X_{t}=x,Z=0|T\geq t\right)}{\hat{P}\left(Z=0|T\geq t\right)}-\frac{P\left(X_{t}=x,Z=0|T\geq t\right)}{P\left(Z=0|T\geq t\right)}\right).$$

It can be readily shown that:

$$\sqrt{n_{t}}\left(\hat{p_{x|0}}-p_{x|0}\right)\;=\frac{1}{\hat{P}\left(Z=0|T\geq t\right)}\sqrt{n_{t}}\left(\hat{P}\left(X_{t}=x,Z=0|T\geq t\right)-P\left(X_{t}=x,Z=0|T\geq t\right)\right)-\frac{P\left(X_{t}=x,Z=0|T\geq t\right)}{\hat{P}\left(Z=0|T\geq t\right)P\left(Z=0|T\geq t\right)}\sqrt{n_{t}}\left(\hat{P}\left(Z=0|T\geq t\right)-P\left(Z=0|T\geq t\right)\right).$$

Let $p_{0}≔P(Z=0|T\geq t)$ and $p_{0x}≔P\left(X_{t}=x,Z=0|T\geq t\right)$. Since $\hat{p_{0}}$ is a consistent estimator of $p_{0}$, we obtain:

$$\sqrt{n_{t}}\left(\hat{p_{x|0}}-p_{x|0}\right)\;\overset{d}{=}\sqrt{n_{t}}\left({𝒫}_{n}-𝒫\right)\left(\frac{1}{p_{0}}\left(I\left(X_{t}=x,Z=0|T\geq t\right)-p_{0x}\right)-\frac{p_{0x}}{p_{0}^{2}}\left(I\left(Z=0|T\geq t\right)-p_{0}\right)\right)$$

as a consequence of Slutsky’s theorem.

*Step 3:* In this step, we combine the above results and conclude the convergence of $\sqrt{n_{t}}(\hat{F}-F)$. We first introduce some notation,

(A1)
$$\eta_{0}=-s_{0}\left(c\right)I\left(Z=0|T\geq t\right)\int_{t}^{c}\frac{dN\left(u\right)-𝒴\left(u\right)d\Lambda_{0}\left(u\right)}{E\left(I\left(Z=0|T\geq t\right)𝒴\left(u\right)\right)},$$

(A2)
$$\eta_{1}=-s_{1}\left(c\right)I\left(Z=1|T\geq t\right)\int_{t}^{c}\frac{dN\left(u\right)-𝒴\left(u\right)d\Lambda_{1}\left(u\right)}{E\left(I\left(Z=1|T\geq t\right)𝒴\left(u\right)\right)},$$

(A3)
$$\eta_{1x}=-s_{1x}\left(c\right)I\left(Z=1,X_{t}=x|T\geq t\right)\int_{t}^{c}\frac{dN\left(u\right)-𝒴\left(u\right)d\Lambda_{1x}\left(u\right)}{E\left(I\left(Z=1,X_{t}=x|T\geq t\right)𝒴\left(u\right)\right)},$$

(A4)
$$\eta_{0x}^{p}=\frac{1}{p_{0}}\left(I\left(X_{t}=x,Z=0|T\geq t\right)-p_{0x}\right)-\frac{p_{0x}}{p_{0}^{2}}\left(I\left(Z=0|T\geq t\right)-p_{0}\right).$$

Combining the above results and apply Slutsky’s theorem, we can write:

$$\sqrt{n_{t}}\left(\hat{F}\left(c\right)-F\left(c\right)\right)=\sqrt{n_{t}}\left({𝒫}_{n}-𝒫\right)(\frac{s_{1}-\sum_{x}p_{x|0}s_{1x}}{{\left(s_{1}-s_{0}\right)}^{2}}\cdot \eta_{0}+\frac{\sum_{x}p_{x|0}s_{1x}-s_{0}}{{\left(s_{1}-s_{0}\right)}^{2}}\cdot \eta_{1}+\frac{1}{s_{0}-s_{1}}\underset{x}{\sum }p_{x|0}\cdot \eta_{1x}+\frac{1}{s_{0}-s_{1}}\underset{x}{\sum }s_{1x}\eta_{0x}^{p})+o_{p}\left(1\right).$$

It follows that $\sqrt{n_{t}}(\hat{F}(c)-F(c))$ converges weakly to a zero-mean Gaussian process with covariance function $E\left\{\zeta (c)\zeta \left(c^{\prime }\right)\right\}$ between time points c and $c^{\prime }$, where:

$$\zeta \left(c\right)=\frac{s_{1}-\sum_{x}p_{x|0}s_{1x}}{{\left(s_{1}-s_{0}\right)}^{2}}\cdot \eta_{0}+\frac{\sum_{x}p_{x|0}s_{1x}-s_{0}}{{\left(s_{1}-s_{0}\right)}^{2}}\cdot \eta_{1}+\frac{1}{s_{0}-s_{1}}\underset{x}{\sum }p_{x|0}\cdot \eta_{1x}+\frac{1}{s_{0}-s_{1}}\underset{x}{\sum }s_{1x}\eta_{0x}^{p}.$$

The covariance function $E\left\{\zeta (c)\zeta \left(c^{\prime }\right)\right\}$ can be consistently estimated by $1/n_{t}\sum_{i=1}^{n_{t}}{\overset{ˆ}{\zeta }}_{i}(c){\overset{ˆ}{\zeta }}_{i}\left(c^{\prime }\right)$ with:

$${\hat{\zeta }}_{i}\left(c\right)=\frac{s_{1}-\sum_{x}p_{x|0}s_{1x}}{{\left(s_{1}-s_{0}\right)}^{2}}\cdot \eta_{0i}+\frac{\sum_{x}p_{x|0}s_{1x}-s_{0}}{{\left(s_{1}-s_{0}\right)}^{2}}\cdot \eta_{1i}+\frac{1}{s_{0}-s_{1}}\underset{x}{\sum }p_{x|0}\cdot \eta_{1xi}+\frac{1}{s_{0}-s_{1}}\underset{x}{\sum }s_{1x}\eta_{0xi^{\prime }}^{p}$$

where $\eta_{0i},\eta_{1i},\eta_{1xi}$, and $\eta_{0xi}^{p}$ is the subject i′s realization of (A1)–(A4), respectively. The specific forms are:

$$\eta_{0i}=-s_{0}\left(c\right)I\left(Z_{i}=0|T_{i}\geq t\right)\int_{t}^{c}\frac{dN_{i}\left(u\right)-Y_{i}\left(u\right)d\Lambda_{0}\left(u\right)}{E\left(I\left(Z_{i}=0|T_{i}\geq t\right)Y_{i}\left(u\right)\right)}$$

$$\eta_{1i}=-s_{1}\left(c\right)I\left(Z_{i}=1|T_{i}\geq t\right)\int_{t}^{c}\frac{dN_{i}\left(u\right)-Y_{i}\left(u\right)d\Lambda_{1}\left(u\right)}{E\left(I\left(Z_{i}=1|T_{i}\geq t\right)Y_{i}\left(u\right)\right)}$$

$$\eta_{1xi}=-s_{1x}\left(c\right)I\left(Z_{i}=1,X_{ti}=x|T_{i}\geq t\right)\int_{t}^{c}\frac{dN_{i}\left(u\right)-Y_{i}\left(u\right)d\Lambda_{1x}\left(u\right)}{E\left(I\left(Z_{i}=1,X_{ti}=x|T_{i}\geq t\right)Y\left(u\right)\right)}$$

$$\eta_{0xi}^{p}=\frac{1}{p_{0}}\left(I\left(X_{ti}=y,Z_{i}=0|T_{i}\geq t\right)-p_{0x}\right)-\frac{p_{0x}}{p_{0}^{2}}\left(I\left(Z_{i}=0|T_{i}\geq t\right)-p_{0}\right).$$

□

## Appendix B. Proof of Theorem 2

**Proof.** The proof consists of two steps. First, we show the sufficiency of Condition C1 for F<1. Second, we show the sufficiency of either Condition C2 or C3 for F>0.

*Step 1:* We first expand AB-BB as $\sum_{x}\left(P\left(T\geq c|X_{t}=x,T\geq t,Z=1\right)-\right.\left.P\left(T\geq c|X_{t}=x,T\geq t,Z=0\right)\right)P\left(X_{t}=x|T\geq t,Z=0\right)$. If Condition C1 is satisfied, we have AB-BB>0. Simple algebra reveals (AA-AB)-(AA-BB)<0. Given AA-BB>0, we conclude:

$$F=\frac{AA-AB}{AA-BB}<1.$$

*Step 2:* If $X_{t}$ in the treatment group is stochastically greater than that in the control group, $E_{A}\left(f\left(X_{t}\right)\right)>E_{B}\left(f\left(X_{t}\right)\right)$ for bounded and increasing function f. When $P(T\geq c|\left.X_{t}=x,T\geq t,Z=1\right)$ is increasing with x, we have $\sum_{x}P\left(T\geq c|X_{t}=x\right.,T\geq t,Z=1)P\left(X_{t}\leq x|T\geq t,Z=1\right)>\sum_{x}P\left(T\geq c|X_{t}=x,T\geq t,Z=1\right)\;P\left(X_{t}\leq x|T\geq t,Z=0\right)$, that is, AA>AB. Use the same argument, if $X_{t}$ in the control group is stochastically greater than that in the treatment group and $P\left(T\geq c|X_{t}=x\right.,T\geq t,Z=1)$ is decreasing with x, we have $-\sum_{x}P\left(T\geq c|X_{t}=x,T\geq t,Z=1\right)\;P\left(X_{t}\leq x|T\geq t,Z=0\right)>-\sum_{x}P\left(T\geq c|X_{t}=x,T\geq t,Z=1\right)P\left(X_{t}\leq x|T\geq t,Z=1\right)$, that is -AB>-AA. Given AA-BB>0, we conclude:

$$F=\frac{AA-AB}{AA-BB}>0.$$

In summary, if Condition C1 and C2 (or C3) are satisfied, the F-measure is bounded within (0,1). □

## Appendix C. Proof of Theorem 3

**Proof.** *Step 1*. In this step, we show P(T(1)≥c∣T(1)≥t,T(0)≥t)=P(T≥c∣T≥t,Z=1). We first write:

$$P\left(T\left(1\right)\geq c|T\left(1\right)\geq t,T\left(0\right)\geq t\right)=\frac{P\left(T\left(1\right)\geq c,T\left(1\right)\geq t|T\left(0\right)\geq t\right)}{P\left(T\left(1\right)\geq t|T\left(0\right)\geq t\right)}.$$

By Assumption 7, we have:

$$P\left(T\left(1\right)\geq c|T\left(1\right)\geq t,T\left(0\right)\geq t\right)=\frac{P\left(T\left(1\right)\geq c,T\left(1\right)\geq t\right)}{P\left(T\left(1\right)\geq t\right)}.$$

Further, Assumption 8 yields:

$$P\left(T\left(1\right)\geq c|T\left(1\right)\geq t,T\left(0\right)\geq t\right)=\frac{P\left(T\left(1\right)\geq c,T\left(1\right)\geq t|Z=1\right)}{P\left(T\left(1\right)\geq t|Z=1\right)}\;=\frac{P\left(T\geq c|Z=1\right)}{P\left(T\geq t|Z=1\right)}\;=P\left(T\geq c|T\geq t,Z=1\right).$$

In a similar way, we can show P(T(0)≥c∣T(1)≥t,T(0)≥t)=P(T≥t∣T≥t,Z=0).

*Step 2*. In this step, we show $P\left(T\left(1,X_{t}=X_{t}(0)\right)\geq c|T(1)\geq t,T(0)\geq t\right)=\sum_{x}P(T\geq c\left.|X_{t}=x,T\geq t,Z=1\right)P\left(X_{t}=x|T\geq t,Z=0\right)$. By definition,

$$P\left(T\left(1,X_{t}=X_{t}\left(0\right)\right)\geq c|T\left(1\right)\geq t,T\left(0\right)\geq t\right)\;=\underset{x}{\sum }P\left(T\left(1,X_{t}=x\right)\geq c,X_{t}\left(0\right)=x|T\left(1\right)\geq t,T\left(0\right)\geq t\right)\;=\underset{x}{\sum }P\left(T\left(1,X_{t}=x\right)\geq c|X_{t}\left(0\right)=x,T\left(1\right)\geq t,T\left(0\right)\geq t\right)P\left(X_{t}\left(0\right)=x|T\left(1\right)\geq t,T\left(0\right)\geq t\right).$$

In the following, we work on the two probability components one by one.

$$P\left(X_{t}\left(0\right)=x|T\left(1\right)\geq t,T\left(0\right)\geq t\right)=\frac{P\left(X_{t}\left(0\right)=x,T\left(0\right)\geq t|T\left(1\right)\geq t\right)}{P\left(T\left(0\right)\geq t|T\left(1\right)\geq t\right)}.$$

The cross-world independence described in Assumption 7 leads to:

$$P\left(X_{t}\left(0\right)=x|T\left(1\right)\geq t,T\left(0\right)\geq t\right)=\frac{P\left(X_{t}\left(0\right)=x,T\left(0\right)\geq t\right)}{P\left(T\left(0\right)\geq t\right)}.$$

Furthermore, Assumption 8 yields:

$$P\left(X_{t}\left(0\right)=x|T\left(1\right)\geq t,T\left(0\right)\geq t\right)=\frac{P\left(X_{t}\left(0\right)=x,T\left(0\right)\geq t|Z=0\right)}{P\left(T\left(0\right)\geq t|Z=0\right)}\;=P\left(X_{t}=x|T\geq t,Z=0\right).$$

The remaining probability component:

$$P\left(T\left(1,X_{t}=x\right)\geq c|X_{t}\left(0\right)=x,T\left(1\right)\geq t,T\left(0\right)\geq t\right)\;=\frac{P\left(T\left(1,X_{t}=x\right)\geq c,T\left(1\right)\geq t|X_{t}\left(0\right)=x,T\left(0\right)\geq t\right)}{PT\left(1\right)\geq t|X_{t}\left(0\right)=x,T\left(0\right)\geq t)}\;=\frac{P\left(T\left(1,X_{t}=x\right)\geq c,T\left(1\right)\geq t\right)}{PT\left(1\right)\geq t)}\;=\frac{P\left(T\left(1,X_{t}=x\right)\geq c,T\left(1\right)\geq t|Z=1\right)}{P\left(T\left(1\right)\geq t|Z=1\right)}\;(\text{by}\;\text{Assumption}\;8).$$

Then we have:

$$P\left(T\left(1,X_{t}=x\right)\geq c|X_{t}\left(0\right)=x,T\left(1\right)\geq t,T\left(0\right)\geq t\right)=P\left(T\left(1,X_{t}=x\right)\geq c|T\geq t,Z=1\right).$$

Assumption 9 gives $X_{t}(1)⊥T\left(1,X_{t}=x\right)\geq c|T\geq t,Z=1$, so that:

$$P\left(T\left(1,X_{t}=x\right)\geq c|X_{t}\left(0\right)=x,T\left(1\right)\geq t,T\left(0\right)\geq t\right)\;=P\left(T\left(1,X_{t}=x\right)\geq c|X_{t}\left(1\right)=x,T\geq t,\text{Z}=1\right)\;=P\left(T\geq c|X_{t}=x,T\geq c,Z=1\right).$$

Collecting the equations for the two probability components, we show:

$$P\left(T\left(1,X_{t}=X_{t}\left(0\right)\right)|T\left(1\right)\geq t,T\left(0\right)\geq t\right)\;=\underset{x}{\sum }P\left(T\geq c|X_{t}=x,T\geq t,Z=1\right)P\left(X_{t}=x|T\geq t,Z=0\right).$$

□

## Appendix D. F-Measure under the Time-Varying Cox-Weibull Model

To facilitate the exploration and understanding of the F-measure, we calculate its true value under a time-varying Cox–Weibull model as an illustrative example. We follow the notation described in the main paper: Z denotes treatment assignment (control = 0; treatment = 1); $X_{t}$ denotes the value of the marker at time t;T denotes the failure time; and c denotes the pre-specified time of interest for survival.

We consider the time-varying Cox–Weibull model:

$$h\left(t\right)=h_{0}\left(t\right)\text{exp}\left(b_{1}Z+b_{2}X_{t}\right),$$

$$h_{0}\left(t\right)=\lambda vt^{v-1},$$

where the marker value satisfies $X_{t}=I\left(t\geq t_{s}\right)$, and $t_{s}$ follows an exponential distribution with mean $\mu_{z}$. The definition of the F-measure reads:

$$F\left(c,t\right)=\frac{P\left(T\geq c|T\geq t,Z=1\right)-\sum_{x}Pr\left(T\geq c|X_{t}=x,T\geq t,Z=1\right)P\left(X_{t}=x|T\geq t,Z=0\right)}{P\left(T\geq c|T\geq t,Z=1\right)-P\left(T\geq c|T\geq t,Z=0\right)}.$$

With the Bayes rule, the conditional survival probability can be written in the form of:

$$P\left(T\geq c|T\geq t,Z=z\right)=\frac{P\left(T\geq c|Z=z\right)}{P\left(T\geq t|Z=z\right)}.$$

In general, for τ∈(0,∞),

(A5)
$$P\left(T\geq \tau |Z=z\right)=P\left(T\geq \tau ,X_{\tau }=1|Z=z\right)+P\left(T\geq \tau ,X_{\tau }=0|Z=z\right).$$

The first term of (A5):

$$P\left(T\geq \tau ,X_{\tau }=1|Z=z\right)=\int_{0}^{\tau }P\left(T\geq \tau |t_{s},Z=z\right)f\left(t_{s}|Z=z\right)dt_{s}\;=\int_{0}^{\tau }\text{exp}\left(-\int_{0}^{t_{s}}\text{exp}\left(b_{1}z\right)h_{0}\left(u\right)du-\int_{0}^{\tau }\text{exp}\left(b_{1}z+b_{2}\right)h_{0}\left(t\right)du\right)\cdot f\left(t_{s}|Z=z\right)dt_{s}.$$

Plugging in $\int_{a}^{b}h_{0}(u)du=\lambda \left(b^{v}-a^{v}\right)$ and the density $f\left(t_{s}|Z=z\right)=1/\mu_{z}\text{exp}\left(-t_{s}/\mu_{z}\right)$ leads to:

(A6)
$$P\left(T\geq \tau ,X_{\tau }=1|Z=z\right)=\int_{0}^{\tau }\text{exp}\left(-e^{b_{1}z}\lambda t_{s}^{v}-e^{b_{1}z+b_{2}}\lambda \left(\tau^{v}-t_{s}^{v}\right)\right)\frac{1}{\mu_{z}}\text{exp}\left(-\frac{t_{s}}{\mu_{z}}\right)dt_{s}.$$

For a general Cox-Weibull model with v>0, there is no closed form formula and we need to refer to a numerical evaluation. Similarly, the second term of (A5):

(A7)
$$P\left(T\geq \tau ,X_{\tau }=0|Z=z\right)=\int_{\tau }^{\infty }P\left(T\geq \tau |t_{s},Z=z\right)f\left(t_{s}|Z=z\right)dt_{s}\;=\int_{\tau }^{\infty }\text{exp}\left(-\int_{0}^{\tau }\text{exp}\left(b_{1}z\right)h_{0}\left(u\right)du\right)\cdot f\left(t_{s}|Z=z\right)dt_{s}\;=\int_{\tau }^{\infty }\text{exp}\left(-e^{b_{1}z}\lambda \tau^{v}\right)\frac{1}{\mu_{z}}\text{exp}\left(-\frac{t_{s}}{\mu_{z}}\right)dt_{s}\;=\text{exp}\left(-\tau^{v}\lambda e^{b_{1}z}-\frac{\tau }{t_{z}}\right).$$

Thus far, with Equations (A6) and (A7), we can calculate P(T≥c∣T≥t,Z=1) and P(T≥c∣T≥t,Z=0).

Next, the adjusted probability in the numerator of the F-measure $\sum_{x}P\left(T\geq c|X_{t}=x\right.,T\geq t,Z=1)P\left(X_{t}=x|T\geq t,Z=0\right)=\sum_{x}P\left(T\geq c|X_{t}=x,T\geq t,Z=1\right)P\left(X_{t}=x|\right.\;T\geq t,Z=0)$. We use Bayes rule and obtain:

(A8)
$$P\left(T\geq c|X_{t}=1,T\geq t,Z=1\right)=\frac{P\left(T\geq c,X_{t}=1|Z=1\right)}{P\left(T\geq t,X_{t}=1|Z=1\right)}.$$

The denominator of Equation (A8) is shown in (A6). The numerator of Equation (A8) satisfies:

$$P\left(T\geq c,X_{t}=1|Z=1\right)=P\left(T\geq c,t_{s}\leq t|Z=1\right)\;=\int_{0}^{t}P\left(T\geq c|t_{s},Z=1\right)f\left(t_{s}|Z=1\right)dt_{s}\;=\int_{0}^{t}\text{exp}\left(-\int_{0}^{t_{s}}\text{exp}\left(b_{1}\right)h_{0}\left(u\right)du-\int_{t_{s}}^{c}\text{exp}\left(b_{1}+b_{2}\right)h_{0}\left(u\right)du\right)\cdot f\left(t_{s}|Z=1\right)dt_{s}$$

Plugging in $\int_{a}^{b}h_{0}(u)du=\lambda \left(b^{v}-a^{v}\right)$ and the density $f\left(t_{s}|Z=z\right)=1/\mu_{z}\text{exp}\left(-t_{s}/\mu_{z}\right)$ leads to:

(A9)
$$P\left(T\geq c,X_{t}=1|Z=1\right)=\int_{0}^{t}\text{exp}\left(-e^{b_{1}}\lambda t_{s}^{v}-e^{b_{1}+b_{2}}\lambda \left(c^{v}-t_{s}^{v}\right)\right)\cdot \frac{1}{\mu_{1}}\text{exp}\left(-\frac{t_{s}}{\mu_{1}}\right)dt_{s}.$$

Following the same lines, we work on:

(A10)
$$P\left(T\geq c|X_{t}=0,T\geq t,Z=1\right)=\frac{P\left(T\geq c,X_{t}=0|Z=1\right)}{P\left(T\geq t,X_{t}=0|Z=1\right)}.$$

The numerator of Equation (A10) reads:

(A11)
$$P\left(T\geq c,X_{t}=0|Z=1\right)=P\left(T\geq c|Z=1\right)-P\left(T\geq c,X_{t}=1|Z=1\right),$$

and the denominator reads:

(A12)
$$P\left(T\geq t,X_{t}=0|Z=1\right)=P\left(T\geq t|Z=1\right)-P\left(T\geq t,X_{t}=1|Z=1\right).$$

To simplify the math, we introduce the following notation:

A(τ,z)≔P(T≥τ,Xτ=1∣Z=z)=∫0τexp(−eb1zλtsv−eb1z+b2λ(τv−tsv))1μzexp(−tsμz)dts,B(τ,z)≔P(T≥τ,Xτ=0∣Z=z)=exp(−τvλeb1z−τμz),C≔P(T≥c,Xt=1∣Z=1)=∫0τexp(−eb1λtsv−eb1+b2λ(cv−tsv))1μ1exp(−tsμ1)dts.

With the above notation, Equation (A5) writes:

P(T≥τ|Z=z)=A(τ,z)+B(τ,z),

Equation (A8) writes:

$$P\left(T\geq c|X_{t}=1,T\geq t,Z=1\right)=\frac{C}{A\left(t,1\right)},$$

and Equation (A10) writes:

$$P\left(T\geq c|X_{t}=0,T\geq t,Z=1\right)=\frac{A\left(c,1\right)+B\left(c,1\right)-C}{A\left(t,1\right)+B\left(t,1\right)-A\left(t,1\right)}=\frac{A\left(c,1\right)+B\left(c,1\right)-C}{B\left(t,1\right)}.$$

The remaining parts in the F-measure definition are:

$$P\left(X_{t}=1|T\geq t,Z=0\right)=\frac{P\left(X_{t}=1,T\geq t|Z=0\right)}{P\left(T\geq t|Z=0\right)}=\frac{A\left(t,0\right)}{A\left(t,0\right)+B\left(t,0\right)},$$

$$P\left(X_{t}=0|T\geq t,Z=0\right)=1-P\left(X_{t}=1|T\geq t,Z=0\right)=\frac{B\left(t,0\right)}{A\left(t,0\right)+B\left(t,0\right)}.$$

Gathering all the pieces together, we have:

$$P\left(T\geq c|T\geq t,Z=1\right)=\frac{A\left(c,1\right)+B\left(c,1\right)}{A\left(t,1\right)+B\left(t,1\right)},$$

$$P\left(T\geq c|T\geq t,Z=0\right)=\frac{A\left(c,0\right)+B\left(c,0\right)}{A\left(t,0\right)+B\left(t,0\right)},$$

$$Pr\left(T\geq c|X_{t}=1,T\geq t,Z=1\right)P\left(X_{t}=1|T\geq t,Z=0\right)=\frac{CA\left(t,0\right)}{A\left(t,1\right)\left(A\left(t,0\right)+B\left(t,0\right)\right)},$$

$$Pr\left(T\geq c|X_{t}=0,T\geq t,Z=1\right)P\left(X_{t}=0|T\geq t,Z=0\right)=\frac{\left(A\left(c,1\right)+B\left(c,1\right)-C\right)B\left(t,0\right)}{B\left(t,1\right)\left(A\left(t,0\right)+B\left(t,0\right)\right)}.$$

When v=1 (i.e., the failure time follows an exponential distribution), terms A and C are equipped with a closed-form formula in the form of:

$$A\left(\tau ,z\right)≔\frac{\text{exp}\left(-\tau \lambda e^{b_{1}z+b_{2}}\right)-\text{exp}\left(-\tau \left(\lambda e^{b_{1}z}+1/\mu_{z}\right)\right)}{1+\lambda \mu_{z}e^{b_{1}z}\left(1-e^{b_{2}}\right)},$$

$$B\left(\tau ,z\right):=\text{exp}\left(-\tau \left(\lambda e^{b_{1}z}+1/t_{z}\right)\right),$$

$$C≔\frac{\text{exp}\left(-c\lambda e^{b_{1}+b_{2}}\right)\left(1-\text{exp}\left(-\frac{t}{\mu_{1}}+\lambda te^{b_{1}}\left(-1+e^{b_{2}}\right)\right)\right)}{1+\lambda \mu_{1}e^{b_{1}}\left(1-e^{b_{2}}\right)}.$$
